# Morphological and phenotypical features of ovarian metastases in breast cancer patients

**DOI:** 10.1186/s12885-017-3191-y

**Published:** 2017-03-21

**Authors:** Inge T. A. Peters, Merle A. van der Steen, Bertine W. Huisman, Carina G. J .M. Hilders, Vincent T. H. B. M. Smit, Alexander L. Vahrmeijer, Cornelis F. M. Sier, J. Baptist Trimbos, Peter J. K. Kuppen

**Affiliations:** 1 Department of Gynecology, Leiden University Medical Center, Leiden, the Netherlands; 2 Department of Surgery, Leiden University Medical Center, Leiden, the Netherlands; 3 Department of Gynecology, Reinier de Graaf Hospital, Delft, the Netherlands; 4 Department of Pathology, Leiden University Medical Center, Leiden, the Netherlands

**Keywords:** breast cancer, fertility preservation, ovarian metastases, ovarian tissue autotransplantation, tumor markers, tumor-specific imaging

## Abstract

**Background:**

Autotransplantation of frozen-thawed ovarian tissue is a method to preserve ovarian function and fertility in patients undergoing gonadotoxic therapy. In oncology patients, the safety cannot yet be guaranteed, since current tumor detection methods can only exclude the presence of malignant cells in ovarian fragments that are not transplanted. We determined the need for a novel detection method by studying the distribution of tumor cells in ovaries from patients with breast cancer. Furthermore, we examined which cell-surface proteins are suitable as a target for non-invasive tumor-specific imaging of ovarian metastases from invasive breast cancer.

**Methods:**

Using the nationwide database of the Dutch Pathology Registry (PALGA), we identified a cohort of 46 women with primary invasive breast cancer and ovarian metastases. The localization and morphology of ovarian metastases were determined on hematoxylin-and-eosin-stained sections. The following cell-surface markers were immunohistochemically analyzed: E-cadherin, epithelial membrane antigen (EMA), human epidermal growth receptor type 2 (Her2/neu), carcinoembryonic antigen (CEA), αvβ6 integrin and epithelial cell adhesion molecule (EpCAM).

**Results:**

The majority of ovarian metastases (71%) consisted of a solitary metastasis or multiple distinct nodules separated by uninvolved ovarian tissue, suggesting that ovarian metastases might be overlooked by the current detection approach. Combining the targets E-cadherin, EMA and Her2/neu resulted in nearly 100% detection of ductal ovarian metastases, whereas the combination of EMA, Her2/neu and EpCAM was most suitable to detect lobular ovarian metastases.

**Conclusions:**

Examination of the actual ovarian transplants is recommended. A combination of targets is most appropriate to detect ovarian metastases by tumor-specific imaging.

**Electronic supplementary material:**

The online version of this article (doi:10.1186/s12885-017-3191-y) contains supplementary material, which is available to authorized users.

## Background

Cryopreservation of ovarian tissue is the only option to preserve fertility and restore ovarian activity in prepubescent girls and women who cannot postpone the start of adjuvant chemotherapy [1]. Although autotransplantation of frozen-thawed cortical ovarian tissue has resulted in more than 86 live births worldwide [2], this method has not yet been endorsed by the American Society for Reproductive Medicine (ASRM) [3]. One of the reasons that the ASRM committee has put forward is that the safety of the procedure has not been substantiated in patients with cancer. Cortical ovarian tissue may contain malignant cells that could lead to reseeding of cancer upon autotransplantation. This risk of reintroducing malignant cells cannot be eliminated, since the current tumor detection methods (e.g. PCR, immunohistochemistry) jeopardize the ovarian tissue’s viability [4]. These methods can therefore only be used to examine cortical ovarian strips that are not transplanted. Hence, the presence of tumor cells in the actual ovarian autografts remains questionable.

Whether the current approach for tumor detection is accurate depends on the distribution of metastatic tumor cells in the ovarian tissue [5, 6]. If tumor cells are diffusely dispersed throughout the ovary, examination of one or two cortical ovarian strips might be sufficient. By contrast, if tumor cells are confined to a specific area in the ovarian cortex, this approach is inadequate. Then, cortical ovarian strips that are examined may turn out to be devoid of tumor cells whereas ovarian fragments that harbor metastases may be transplanted, possibly resulting in cancer relapse.

The implementation of a detection method that allows examination of the cortical ovarian strips that will be transplanted, will significantly reduce the risk of transferring malignant cells. Near-infrared fluorescence (NIRF) imaging might be an appropriate approach, as this technique discriminates malignant cells from non-malignant tissue in real time while leaving the tissues viable [7]. A NIRF probe consists of a fluorophore that emits light in the near-infrared spectrum (λ = 700–900 nm) and an antibody or peptide with high affinity for a protein expressed specifically at the cell surface of tumor cells [8, 9].

In order to use tumor-specific imaging to exclude malignant cells in cortical ovarian autografts, tumor markers should be identified that are present at the cell surface of ovarian metastases. Since a substantial proportion of patients who undergo ovarian tissue cryopreservation is diagnosed with breast cancer [10–12], we tested a panel of cell-surface markers known to be expressed by breast cancer cells, including E-cadherin [13], epithelial membrane antigen (EMA, also known as MUC1) [14, 15], human epidermal growth factor receptor type 2 (Her2/neu) [16, 17], carcinoembryonic antigen (CEA) [18], αvβ6 integrin [19] and epithelial cell adhesion molecule (EpCAM) [20–22]. The markers cytokeratin CAM 5.2, gross cystic disease fluid protein-15 (GCDFP15), Wilms’ tumor antigen-1 (WT1), mammaglobin 1, and cytokeratin 7 (CK-7), which were used by Sánchez-Serrano et al. [23] and Rosendahl et al. [6], were excluded, as they are not expressed at the cell surface and therefore not suitable as a target for tumor-specific imaging.

In this study, we assessed the distribution of breast tumor cells in ovarian tissues from patients with ovarian metastases and determined which cell-surface proteins are suitable as a target for tumor-specific imaging of ovarian metastases derived from invasive breast cancer. Because it is crucial to select a target prior to the administration of the NIRF probe, we also examined whether invasive breast cancer tissue can be used to predict the most suitable target for the detection of ovarian metastases in a particular patient.

## Methods

### Patient selection and tissue collection

Via a nationwide search performed by PALGA, the Dutch histopathology and cytopathology network that encompasses all pathology laboratories within the Netherlands [24], a source population was compiled. This source population consisted of all patients who were diagnosed with primary invasive breast cancer at age < 41 years in the period 2000–2010 and who subsequently underwent an oophorectomy for any reason. From this source population, all patients who had histologically confirmed ovarian metastases from primary invasive breast cancer, were selected. Following this, hematoxylin-and-eosin (H&E) stained tissue sections and formalin-fixed paraffin-embedded (FFPE) tissue samples from the primary invasive breast tumors and their corresponding ovarian metastases were requested from pathology laboratories. If patients had locally recurrent breast cancer or a second primary invasive breast tumor prior to oophorectomy, FFPE tissue samples from these tumors were also requested. Clinical data were extracted from the patient’s files after approval by the medical ethical committee of the Leiden University Medical Center (protocol number P14.106) and the local medical ethical committees of the participating hospitals.

### Distribution of breast cancer cells in the ovary

The distribution of breast cancer cells in ovarian tissues was evaluated using the original H&E-stained sections by assessment of their localization and morphological features. The localization of breast cancer cells was determined as confined to the ovarian cortex and/or medulla. With respect to morphology, breast cancer cells were classified as a solitary metastasis, multiple distinct nodules separated by uninvolved ovarian tissue, or diffuse seeding without any discernable pattern.

### Immunohistochemistry

Immunohistochemistry was performed on 4-μm thick FFPE sections of primary invasive breast cancers, locally recurrent breast cancers (if applicable) and their corresponding ovarian metastases. The tissue sections were deparaffinized in xylene, dehydrated in a stepwise series of graded alcohol solutions, and rinsed in distilled water. After blocking endogenous peroxidase activity with 0.3% hydrogen peroxide for 20 min, heat-induced antigen retrieval was performed by placing the slides in EnVision Flex Target Retrieval Solution high pH (pH 9.0; E-cadherin, EMA) or in the same solution but low pH (pH 6.0; Her2/neu) in PT Link (Dako, Denmark). EpCAM and αvβ6 integrin epitopes were unmasked by 30-min incubation with 0.125% trypsin and 0.4% pepsin, respectively, at 37 °C. For CEA, no antigen retrieval was required. The sections were incubated overnight in a humidified chamber at room temperature with primary antibodies against Her2/neu (ERBB2, rabbit polyclonal, Dako), E-cadherin (NCH38, mouse monoclonal, Dako), EpCAM (323/A3, mouse monoclonal, provided by the Department of Pathology, LUMC, the Netherlands), CEA (A0115, rabbit polyclonal, Dako), αvβ6 integrin (6.2A1, mouse monoclonal, Cell Essentials), or EMA (E29, mouse monoclonal, Dako); all primary antibodies were used at their predetermined optimal dilution. After incubation with primary antibodies, the sections were rinsed with PBS, incubated with secondary antibodies (anti-mouse or anti-rabbit EnVision; Dako) for 30 min, and visualized using liquid DAB+ substrate buffer (Dako). The sections were counterstained with Mayer’s hematoxylin solution, dehydrated, and mounted with Pertex (Leica Microsystems, Germany). For each immunostain, tissues expressing the antigen of interest were included as a positive control. Tissue sections stained without application of the primary antibody were used as a negative control.

### Immunofluorescent triple staining

For immunofluorescent triple staining, the three most highly expressed markers for ductal and lobular ovarian metastases were chosen. In brief, FFPE sections of these ovarian metastases were deparaffinized as described above. Antigen retrieval was performed by placing the slides in EnVision Flex Target Retrieval Solution high pH (pH 9.0; Dako). Primary antibodies for ductal ovarian metastases: E-cadherin, EMA and Her2/neu. Primary antibodies for lobular ovarian metastases: EMA, Her2/neu and EpCAM. Secondary antibodies were all isotype-specific antibodies with Alexa Fluorochromes (LifeTechnologies, USA): anti-mouse IgG1-AlexaFluor488 (E-cadherin and EpCAM; green), anti-mouse IgG2a-AlexaFluor647 (EMA; red) and anti-rabbit-AlexaFluor546 (Her2/neu; orange). Sections were mounted with Vectashield containing DAPI (Vector Laboratories, USA). Primary invasive breast tumor samples that showed positive expression for all markers in previous experiments were used as a positive control. Tissue sections stained without application of primary antibodies were used as a negative control.

### Image capture and quantification of immunoreactivity

The immunohistochemically stained slides were digitized using an IntelliSite Pathology Ultra-Fast Scanner 1.6 RA (Philips, The Netherlands). The percentage of malignant cells with immunohistochemically positive stained membranes were scored by two independent observers (I.P. and M.S.). In case of discrepancy, the observers reached consensus regarding a final score. The tumor cell membranes were considered positive if they showed immunoreactivity of any intensity. The immunofluorescent stained slides were digitized using a Pannoramic MIDI digital slide scanner (3DHistech, Hungary). The percentage of malignant cells with immunofluorescent positive stained membranes were also scored by two independent observers (I.P. and B.H.).

### Statistical analysis

Statistical analysis was performed using SPSS version 23.0 (IBM, Armonk, NY). Inter-observer agreement was calculated using the Pearson correlation coefficient. Scatter plots based on generalized estimating equations analysis were made to determine whether invasive breast cancer tissue can be used to predict the most suitable target for the detection of ovarian metastases in a particular patient.

## Results

### Patient selection and clinicopathological characteristics

**Fig. 1:**
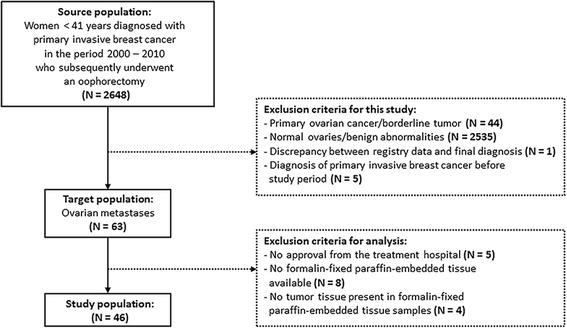
Patient selection and composition of the study population. The source population was compiled by the Dutch histopathology and cytopathology network. The exclusion criteria are indicated in the dotted boxes

According to the PALGA registry, 2648 patients were diagnosed with primary invasive breast cancer at age < 41 years in the period 2000–2010 in the Netherlands who subsequently underwent an oophorectomy (Fig. 1). Among these patients, 63 patients had ovarian metastases. Of these 63 patients, tumor tissue samples were available from 46 patients. These 46 patients were included in this study. The clinicopathological characteristics of the 46 patients are shown in Table 1. The median age at the time of diagnosis was 36.5 years (range 28–40 years). Thirty-six patients were diagnosed with invasive ductal breast cancer and five patients were diagnosed with invasive lobular breast cancer. The remaining five patients had invasive ductolobular breast cancer. Almost 15% of patients had distant metastases outside the ovary at the time of breast cancer diagnosis. The median time between this diagnosis and oophorectomy was 41.9 months (range 0.3–141.8 months). In the majority of cases, the oophorectomy was done prophylactically or therapeutically because of breast cancer. In only one fourth of cases, the ovaries were removed because they appeared abnormal on ultrasound. Further patient and tumor characteristics are presented in Table 1.

**Table 1:** Clinicopathological characteristics of patients with primary invasive breast cancer and ovarian metastases

Clinicopathological characteristics	*N* = 46	%
Age at diagnosis of breast cancer, years - median (range)	36.5 (28–40)	-
*BRCA* gene mutation		
No	8	17.4
Yes, *BRCA* 1	1	2.2
Yes, *BRCA* 2	0	0.0
Unknown	37	80.4
Breast tumor localization		
Left	23	50.0
Right	21	45.7
Both	2	4.3
Most extensively performed breast surgery		
Needle biopsy	4	8.7
Breast conserving surgery	15	32.6
Mastectomy	27	58.7
Breast tumor histological subtype		
Ductal	36	78.2
Lobular	5	10.9
Ductolobular	5	10.9
Scarff-Bloom-Richardson grade		
I	4	8.7
II	19	41.3
III	15	32.6
Unknown	8	17.4
Estrogen receptor		
Negative	5	10.9
Positive	41	89.1
Progesterone receptor		
Negative	8	17.4
Positive	38	82.6
Her2/neu receptor		
Negative	38	82.6
Positive	8	17.4
Tumor stage		
T1	11	23.9
T2	24	52.2
T3	7	15.2
T4	4	8.7
Nodal stage		
N0	14	30.4
N1	12	26.1
N2	10	21.7
N3	10	21.7
Distant metastasis		
cM0	39	84.8
cM1	7	15.2
Age at diagnosis of ovarian metastases, years - median (range)	40.0 (31–51)	-
Time between breast cancer and ovarian metastases, months - median (range)	41.9 (0.3–141.8)	-
Recurrent disease prior to oophorectomy		
No	15	32.6
Yes, locoregional recurrence	12	26.1
Yes, distant recurrence	19	41.3
Type of ovarian surgery		
Unilateral oophorectomy	0	0.0
Bilateral oophorectomy	46	100.0
Indication for oophorectomy		
Prophylactic because of breast cancer	9	19.6
Therapeutic because of breast cancer	25	54.3
Abnormal ovaries on ultrasound	12	26.1
Localization of ovarian metastases		
Left	4	8.7
Right	6	13.0
Both	29	63.0
Unknown	7	15.2

### Localization and morphology of ovarian metastases

**Fig. 2:**
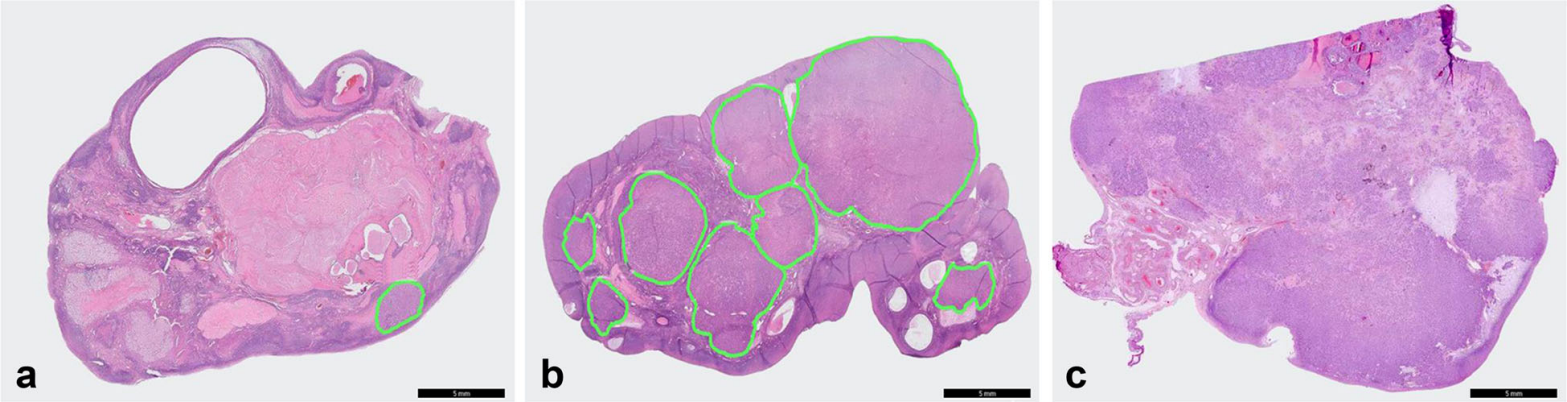
Localization of ovarian metastases derived from patients diagnosed with invasive breast cancer. Three examples are shown: (**a**) a solitary metastasis, (**b**) multiple distinct nodules separated by uninvolved ovarian tissue and (**c**) diffuse seeding without any discernable pattern. In order to clearly display the solitary metastasis in (**a**) and the multiple distinct nodules in (**b**), a green line is drawn that delineates the metastases in the ovary. Scale bars represent 5 mm

Of the 46 patients, 29 patients had metastases in both ovaries (Table 1). Therefore, the total number of ovaries that contained metastases was 75. The localization and morphology of these 75 ovarian metastases are shown in Table 2. In 14 ovaries (19%) the metastases seemed confined to the cortex, whereas in 53 ovaries (70%) both the cortex and medulla were involved (Table 2). In half of the ovaries multiple distinct nodules were seen, while in 20% a solitary metastasis was found. Diffuse seeding without any discernable pattern was observed in 29% of ovaries. Figure 2 shows examples of these morphological features.

**Table 2:** Localization and morphology of ovarian metastases derived from patients diagnosed with invasive breast cancer

Histological features	Ovarian metastases	
	*N* = 75	%
Localization of ovarian metastases		
Cortex	14	18.7
Medulla	8	10.7
Both	53	70.1
Morphology of ovarian metastases		
Solitary metastasis	15	20.0
Multiple distinct nodules separated by uninvolved ovarian tissue	38	50.7
Diffuse seeding without any discernable pattern	22	29.3
Fallopian tube involved		
No	55	73.3
Yes	5	6.7
Unknown	15	20.0

Of the 46 patients who were diagnosed with invasive breast cancer and ovarian metastases, 29 patients had metastases in both ovaries. The total number of ovaries that contained metastases was 75

### Expression of cell-surface proteins

Immunohistochemistry was performed to determine which cell-surface proteins are suitable as a target for tumor-specific imaging of ovarian metastases from invasive breast cancer. A strong correlation was observed between the scoring results obtained by the two observers; the median R^2^ was 0.846 (range: 0.640–0.960). Representative examples of the immunohistochemical stainings of the invasive breast tumor samples and their corresponding ovarian metastases are shown in Additional file 1: Figure S1.

Table 3 shows the mean percentage of positive tumor cells for the investigated markers in primary and recurrent invasive breast tumors and their ovarian metastases. Since loss of expression of the cell-adhesion molecule E-cadherin frequently occurs in invasive lobular carcinomas [25], the expression of markers was examined by histological subtype. With respect to invasive ductal carcinomas, E-cadherin, EMA and Her2/neu were most suitable; these markers were present in 91, 84 and 81% of metastatic breast tumor cells in the ovaries, respectively. In invasive lobular carcinomas, the mean percentage of positively stained breast tumor cells in the ovaries was highest for EMA, Her2/neu and EpCAM; specifically, 64, 74 and 68%, respectively. In patients diagnosed with ductolobular breast cancer, targeting EMA would result in the detection of 99% of disseminated breast cancer cells in the ovaries.

**Table 3:** Immunohistochemical expression of the investigated markers in invasive breast tumors and their corresponding ovarian metastases

Marker	% of positive tumor cells in invasive ductal carcinoma				% of positive tumor cells in invasive lobular carcinoma				% of positive tumor cells in invasive ductolobular carcinoma			
	Breast tumors (*n* = 44)		Ovarian metastases (*n* = 58)		Breast tumors (*n* = 7)		Ovarian metastases (*n* = 10)		Breast tumors (*n* = 7)		Ovarian metastases (*n* = 7)	
	*Mean*	*SD*	*Mean*	*SD*	*Mean*	*SD*	*Mean*	*SD*	*Mean*	*SD*	*Mean*	*SD*
E-cadherin	91	18	91	20	9	23	0	0	73	35	51	41
EMA	86	23	84	24	86	32	64	32	97	6	99	2
Her2/neu	76	35	81	31	88	26	74	26	80	34	67	38
CEA	56	40	57	39	73	32	59	26	62	32	56	33
αvβ6 integrin	51	40	45	39	54	35	38	28	45	30	29	35
EpCAM	36	42	38	39	38	46	68	26	19	29	35	29
SD = standard deviation												

The mean percentages of immunohistochemically positive stained tumor cells are subdivided by histological subtype. Tumor cell membranes were considered positive if they showed immunoreactivity of any intensity. EMA, epithelial membrane antigen; Her2/neu, human epidermal growth receptor type 2; CEA, carcinoembryonic antigen; EpCAM, epithelial cell adhesion molecule

### Correlation between the expression of cell-surface proteins in invasive breast tumors and their corresponding ovarian metastases

**Fig. 3:**
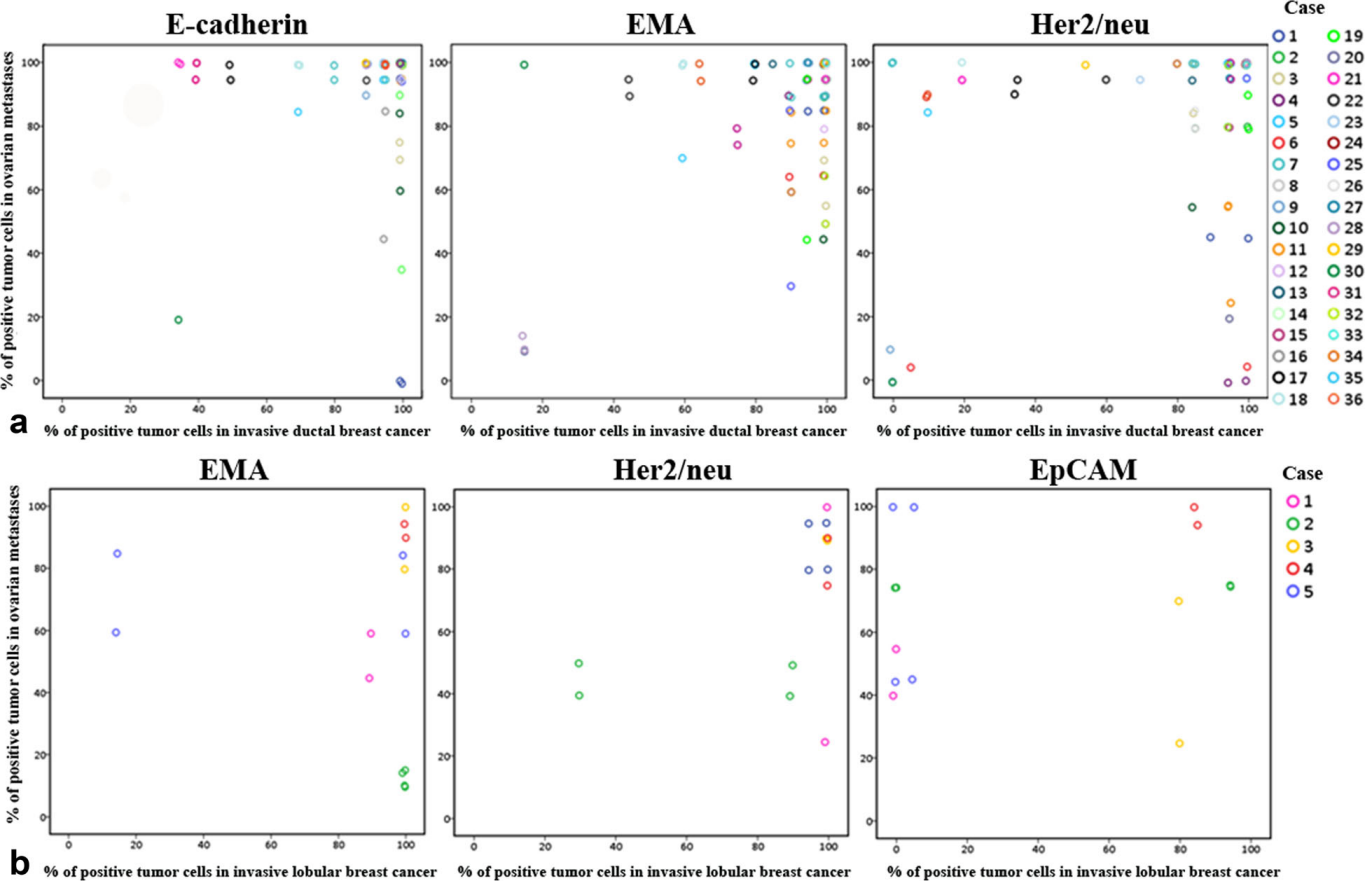
The correlation between tumor marker expression in breast tumors and ovarian metastases for individual patients. Upper panel (**a**) shows invasive ductal breast cancer and lower panel (**b**) represents invasive lobular breast cancer. For each patient, the percentage of positive tumor cells in primary and locally recurrent breast tumors (if applicable) was set against the percentage of positive tumor cells in their corresponding ovarian metastases. EMA, epithelial membrane antigen; Her2/neu, human epidermal growth receptor type 2; EpCAM, epithelial cell adhesion molecule

In patients diagnosed with ductolobular breast cancer, the expression of EMA in the invasive breast tumors was in accordance with the expression in their corresponding ovarian metastases, showing small standard deviations (Table 3). Therefore, EMA would be the most suitable target to detect ductolobular ovarian metastases. By contrast, in patients diagnosed with ductal or lobular breast cancer large variations in expression among tumors were found. To understand whether in these patients invasive breast tumor tissues can be used to predict the most suitable target for the detection of ovarian metastases in an individual patient, scatter plots were made (Fig. 3). For each patient, the percentage of positive tumor cells in primary and locally recurrent breast tumors (if applicable) was set against the percentage of positive tumor cells in their corresponding ovarian metastases. No correlation between these expressions could be substantiated, showing that ductal and lobular breast tumor tissues cannot be used to predict the most pertinent marker for the detection of their corresponding ovarian metastases.

### Detection of ovarian metastases by a combination of markers

**Fig. 4:**
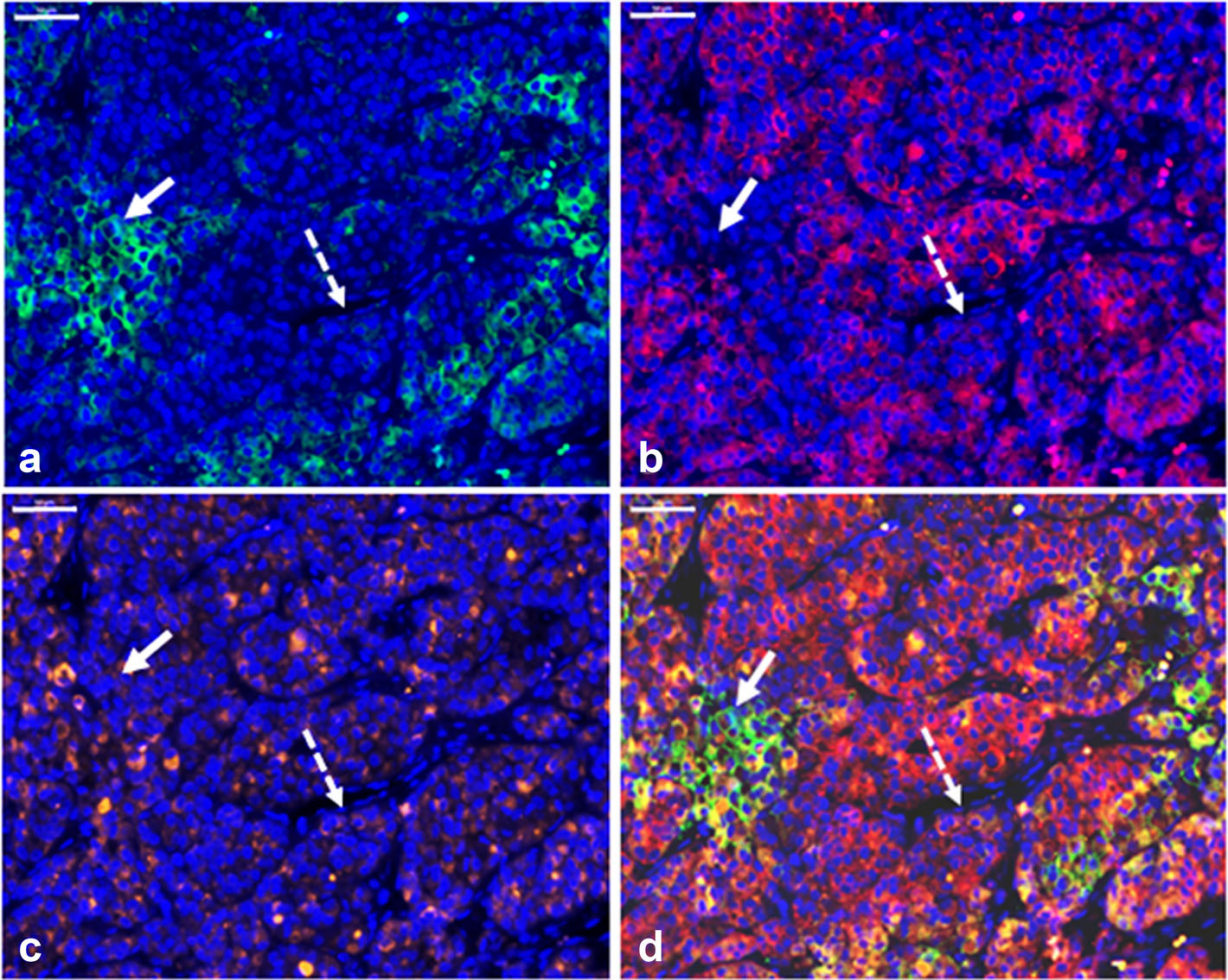
Detection of ovarian metastases by a combination of markers. Representative image of a lobular ovarian metastasis stained with DAPI counterstain and triple immunofluorescence for EpCAM (**a**), EMA (**b**), Her2/neu (**c**), and the three stainings combined (**d**). The solid arrow indicates tumor cells that are positive for EpCAM, but negative for EMA and Her2/neu. The dashed arrow indicates tumor cells that are positive for Her2/neu, but negative for EpCAM and EMA. Scale bars represent 100 μm. EpCAM, epithelial cell adhesion molecule; EMA, epithelial membrane antigen; Her2/neu, human epidermal growth receptor type 2

Figure 3 also shows that the use of one marker would not always be sufficient to detect all metastatic ductal or lobular breast cancer cells in the ovaries. The use of one marker (E-cadherin, EMA or Her2/neu) would result in the detection of 100% of tumor cells in 44 out of 58 ductal ovarian metastases (data not shown). With respect to the lobular subtype, EMA, Her2/neu or EpCAM was present in 100% of tumor cells in 4 out of 10 ovarian metastases. To investigate whether a combination of markers would enable the detection of 100% of tumor cells in all ductal and lobular ovarian metastases, an immunofluorescent triple staining was performed. By combining the three most suitable markers for the ductal (E-cadherin, EMA and Her2/neu) and lobular (EMA, Her2/neu and EpCAM) subtypes, 100% tumor cell detection was accomplished in 53 out of 58 ductal ovarian metastases and in 7 out of 10 lobular ovarian metastases. Hence, cells within ovarian tissues that show membranous positivity for any of the three markers mentioned will be deemed malignant. In the remaining five ductal and three lobular ovarian metastases, the mean percentage of undetected metastatic cells was 5% (no range) and 25% (range 10–40), respectively. Figure 4 shows a representative image of the immunofluorescent triple staining in a lobular ovarian metastasis, in which the combination of EpCAM, EMA and Her2/neu led to the detection of all metastatic breast cancer cells.

## Discussion

One of the purposes of the present study was to examine the histological features of ovarian metastases in breast cancer patients to evaluate the current tumor detection approach [4] in ovarian tissues considered for autotransplantation. We found that 71% of ovarian metastases consisted of a solitary metastasis or multiple distinct nodules separated by uninvolved ovarian tissue. These findings suggest that tumor cells might have been missed if the current tumor detection approach would have been used. The patients included in this study however, underwent oophorectomy after a median time interval of 42 months. In patients undergoing ovarian tissue cryopreservation an oophorectomy is performed soon after cancer diagnosis. In these patients, disseminated tumor cells may not yet have outgrown into overt metastases and may appear as micrometastases in the ovarian tissues [23, 26, 27]. The chance that tumor cells will then be overlooked is presumably greater. We therefore recommend examination of the actual ovarian autografts on the presence of malignant cells prior to autotransplantation.

In our study, merely 63 out of 2648 patients (2.4%) who were diagnosed with primary invasive breast cancer at age < 41 years had ovarian metastases. For the determination of suitable targets for NIRF imaging, it might have been relevant to focus on malignancies with a higher risk of ovarian contamination in the actual patient population, for instance leukemia [28–30]. Nonetheless, breast cancer can be perfectly used as a starting point to investigate whether NIRF imaging is feasible for the detection of ovarian metastases.

Considering the expression of Her2/neu in primary invasive breast cancers and ovarian metastases a high percentage of Her2/neu positive tumor cells (67–88%) was found, as we considered tumor cell membranes positive if they showed immunoreactivity of any intensity. This is in contrast to the diagnostic setting, where Her2/neu overexpression is determined because of its potential prognostic value [17, 31]. We applied a lower cut-off point, because for NIRF imaging the staining intensity is less important as long as a significant tumor-to-background-ratio can be achieved. In the NIR spectrum, non-specific fluorescence background signal is substantially decreased compared to wavelengths lower than NIR [8]. Hence, since ovarian stromal cells do not immunohistochemically express Her2/neu [32], Her2/neu-targeting NIRF probes will detect metastatic breast cancer cells within ovarian tissues if these cells show immunohistochemical reactivity.

In individual patients, no correlation was found between the expression of the investigated markers in breast tumors and their corresponding metastases in the ovary. This might be due to the fact that breast cancer is known as a heterogeneous disease [17] or be in line with the hypothesis that disseminated tumor cells autonomously evolve from the primary tumor [33]. For the clinical application of these markers there should not be an obstacle, since a combination of three markers enhances the ability to detect breast tumor cells in ductal and lobular ovarian metastases. Furthermore, only the histological subtype of the invasive breast tumor needs to be known to determine which combination of markers is pertinent for the detection of the corresponding ovarian metastases, making the selection of suitable NIRF probes simple and straightforward. For the non-invasive detection of metastases in the actual ovarian autografts by tumor-specific imaging, NIRF probes could be administered intravenously, after which the removed ovary is dissected into cortical ovarian strips. Subcellular detailed fluorescent images of tumor cells within ovarian autografts could then be obtained by multiphoton microscopy [34]. Beside breast cancer cells, inclusion cysts will likely also be illuminated by NIRF imaging, as we previously showed that in normal ovaries, all markers (except CEA) were expressed on epithelial cells in inclusion cysts [32]. Nevertheless, we additionally demonstrated that full-field optical coherence tomography (FF-OCT), which creates histology-like images without the need for tissue manipulation, can be perfectly used to differentiate between inclusion cysts and metastases in the ovary [35]. On a tomographic FF-OCT image, an inclusion cyst is characterized by a thin dark outer layer and lack of interior structure, whereas micrometastatic lesions from primary invasive ductal carcinomas present as ‘web-like’ structures in which tumor cells appear light gray. Metastatic lesions derived from primary invasive lobular carcinomas often show an Indian file pattern, defined as infiltrating single rows of cells [36]. Since ovarian inclusion cysts are separately identifiable within the ovarian parenchyma [32], a distinction between these structures can also be made. In addition, FF-OCT and NIRF imaging might be combined to enhance their sensitivity and specificity rates, as both methods are noninvasive. The a priori probability that other benign epithelial ovarian abnormalities will be detected by our panel of cell-surface markers is low, since ovaries that present as an adnexal mass on preoperative ultrasonography are generally not used for ovarian tissue cryopreservation. In case primary ovarian cancer cells are present, these cells will be detected as E-cadherin [37], EMA [38], and EpCAM [39] are virtually always expressed in ovarian cancer, and approximately 33% of primary ovarian carcinomas show Her2/neu amplification [40].

## Conclusions

In conclusion, we showed that in young breast cancer patients with ovarian metastases, metastatic breast tumor cells may be confined to a specific area in the ovarian cortex. A non-invasive tumor detection technique by which cortical ovarian fragments that are transplanted can be examined, is recommended to minimize the risk of reintroducing metastatic tumor cells by ovarian tissue autotransplantation in breast cancer patients. NIRF imaging is a promising technique to discriminate malignant from benign tissues while leaving the examined tissues vital. Our research opens a new avenue for the development of tumor-specific NIRF probes that can be used for non-invasive detection of breast cancer metastases in ovarian tissues prior to autotransplantation.

## Additional files

Additional file 1: Figure S1. Immunohistochemical expression of tumor markers in invasive breast tumors and their corresponding ovarian metastases. Arrows indicate tumor cells that show heterogeneous expression of markers. Scale bars represent 100 μm. EMA, epithelial membrane antigen; Her2/neu, human epidermal growth receptor type 2; CEA, carcinoembryonic antigen; EpCAM, epithelial cell adhesion molecule. (TIFF 5316 kb)

Additional file 2: Table S2. List of participating hospitals. An overview is given of the treatment hospitals that approved the study design. (DOCX 14 kb)

## Abbreviations

ASRM: American Society for Reproductive Medicine
CEA: Carcinoembryonic antigen
CK-7: Cytokeratin 7
DAB: Diaminobenzidine
DAPI: 4′,6-diamidino-2-phenylindole
EMA: Epithelial membrane antigen
EpCAM: Epithelial cell adhesion molecule
FFPE: Formalin-fixed paraffin-embedded
FMWV: Dutch Federation of Biomedical Scientific Societies
GCDFP15: Gross cystic disease fluid protein-15
H&E: Hematoxylin-and-eosin
Her2/neu: Human epidermal growth receptor type 2
NIRF: Near-infrared fluorescence
PALGA: the Dutch Pathology Registry
PBS: Phosphate buffered saline
PCR: Polymerase chain reaction
pH: Potential hydrogen
PT: Pre-treatment
SPSS: Statistical package for the social sciences
WT1: Wilms’ tumor antigen-1
