# Supplementary figures and images for: Morphological and phenotypical features of ovarian metastases in breast cancer patients
Source: BMC Cancer. 2017 Mar 21;17:206. doi: 10.1186/s12885-017-3191-y (PMC5361796; doi:10.1186/s12885-017-3191-y)

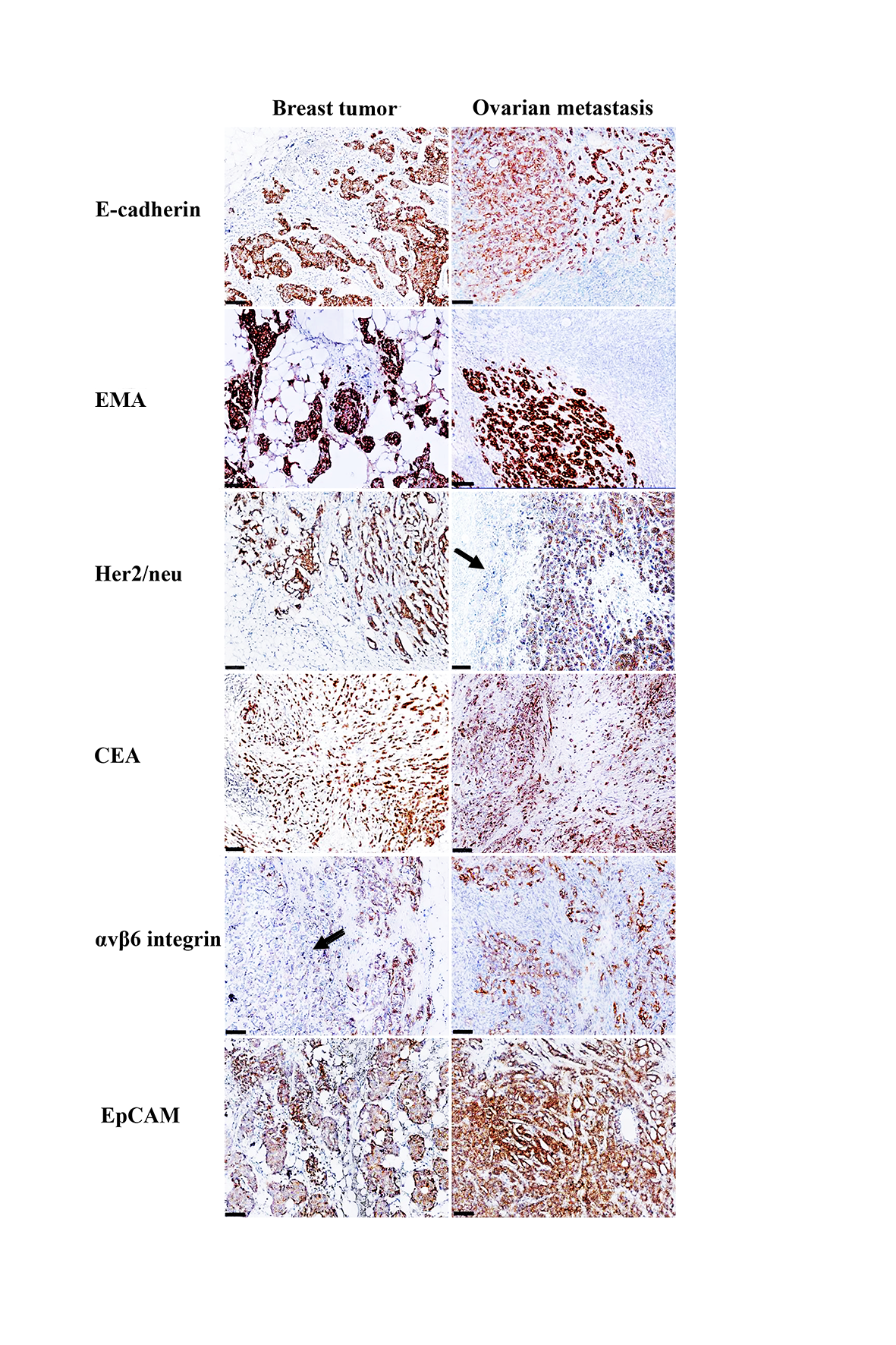

Supplement: Supplementary file 1 — Immunohistochemical expression of tumor markers in invasive breast tumors and their corresponding ovarian metastases. Arrows indicate tumor cells that show heterogeneous expression of markers. Scale bars represent 100 μm. EMA, epithelial membrane antigen; Her2/neu, human epidermal growth receptor type 2; CEA, carcinoembryonic antigen; EpCAM, epithelial cell adhesion molecule. (TIFF 5316 kb) [file 12885_2017_3191_MOESM1_ESM.tif]
