# Supplementary material for: Morphological and phenotypical features of ovarian metastases in breast cancer patients
Source: BMC Cancer. 2017 Mar 21;17:206. doi: 10.1186/s12885-017-3191-y (PMC5361796; doi:10.1186/s12885-017-3191-y)
Supplement: Supplementary file 2 — List of participating hospitals. An overview is given of the treatment hospitals that approved the study design. (DOCX 14 kb) [file 12885_2017_3191_MOESM2_ESM.docx]

**Additional file 2: Table S2. List of participating hospitals.** An overview is given of the treatment hospitals that approved the study design.

| Amphia hospital |
| --- |
| Antoni van Leeuwenhoek hospital - Netherlands Cancer Institute |
| Diakonessenhuis Utrecht |
| Gelre hospital |
| Haga hospital |
| IJsselland hospital |
| Isala klinieken |
| Jeroen Bosch hospital |
| Leiden University Medical Center |
| Lievensberg hospital |
| Maastricht University Medical Center |
| Martini hospital |
| Meander Medical Center |
| Medisch Spectrum Twente |
| Onze Lieve Vrouwen Gasthuis |
| Rijnstate hospital |
| St. Anna hospital |
| St. Antonius hospital |
| St. Lucas Andreas hospital |
| St. Franciscus gasthuis |
| University Medical Center Groningen |
| University Medical Center Utrecht |
| Vlietland hospital |
| VU Medical Center |
| Zaans Medical Center |
| Ziekenhuisgroep Twente |
| Zuiderzee Medical Center |
